# Clinical course and risk factors of fatal adverse outcomes in COVID-19 patients in Korea: a nationwide retrospective cohort study

**DOI:** 10.1038/s41598-021-89548-y

**Published:** 2021-05-12

**Authors:** Juhyun Song, Dae Won Park, Jae-hyung Cha, Hyeri Seok, Joo Yeong Kim, Jonghak Park, Hanjin Cho

**Affiliations:** 1 Department of Emergency Medicine, Korea University Ansan Hospital, Ansan, Republic of Korea; 2 Medical Science Research Centre, Korea University Ansan Hospital, Ansan, Republic of Korea; 3 Division of Infectious Diseases, Department of Internal Medicine, Korea University Ansan Hospital, Ansan, Republic of Korea

**Keywords:** Epidemiology, Risk factors

## Abstract

We investigated association between epidemiological and clinical characteristics of coronavirus disease 2019 (COVID-19) patients and clinical outcomes in Korea. This nationwide retrospective cohort study included 5621 discharged patients with COVID-19, extracted from the Korea Disease Control and Prevention Agency (KDCA) database. We compared clinical data between survivors (n = 5387) and non-survivors (n = 234). We used logistic regression analysis and Cox proportional hazards model to explore risk factors of death and fatal adverse outcomes. Increased odds ratio (OR) of mortality occurred with age (≥ 60 years) [OR 11.685, 95% confidence interval (CI) 4.655–34.150, *p* < 0.001], isolation period, dyspnoea, altered mentality, diabetes, malignancy, dementia, and intensive care unit (ICU) admission. The multivariable regression equation including all potential variables predicted mortality (AUC = 0.979, 95% CI 0.964–0.993). Cox proportional hazards model showed increasing hazard ratio (HR) of mortality with dementia (HR 6.376, 95% CI 3.736–10.802, *p* < 0.001), ICU admission (HR 4.233, 95% CI 2.661–6.734, *p* < 0.001), age ≥ 60 years (HR 3.530, 95% CI 1.664–7.485, *p* = 0.001), malignancy (HR 3.054, 95% CI 1.494–6.245, *p* = 0.002), and dyspnoea (HR 1.823, 95% CI 1.125–2.954, *p* = 0.015). Presence of dementia, ICU admission, age ≥ 60 years, malignancy, and dyspnoea could help clinicians identify COVID-19 patients with poor prognosis.

## Introduction

Since the coronavirus disease 2019 (COVID-19) outbreak in Wuhan, China, COVID-19 has become a major global health problem. Clusters of COVID-19 pneumonia cases led to the eventual identification of severe acute respiratory syndrome coronavirus 2 (SARS-CoV-2). The World Health Organization (WHO) declared COVID-19 a pandemic on March 11, 2020. As of December 29, 2020, there were 81,278,104 confirmed cases from 220 countries, with 1,774,388 deaths, and an overall projected case fatality rate of 2.2%^1^.

The COVID-19 pandemic has led to shortages of medical resources, including hospital beds, ventilators, and personal protective equipment in several countries^2–4^. Therefore, accurate diagnosis and outcome prediction are essential to decrease the burden on national healthcare systems and provide optimal care for COVID-19 patients.

During the early period of the outbreak, some case series from China reported the epidemiological and clinical characteristics of COVID-19^5–7^. Since then, some cohort studies have described potential predictors of mortality and poor clinical outcomes among patients with COVID-19^8–10^. Recently, some large cohort studies including nationwide data (United Kingdoms, Germany and France, respectively) reported the clinical characteristics and factors associated with outcomes among COVID-19 patients^11–13^. Although this nationwide COVID-19 evidence has emerged, there are still controversies about risk factors of mortality and poor clinical outcomes among COVID-19 patients.

By using the data collected by the Korea Disease Control and Prevention Agency (KDCA), we presented details of all patients with laboratory-confirmed COVID-19 and a definite clinical outcome (survival discharge or death) as of April 30, 2020. We aimed to explore the risk factors of mortality and fatal adverse outcomes among COVID-19 patients in Korea.

## Results

### Demographic and clinical characteristics

**Figure 1:**
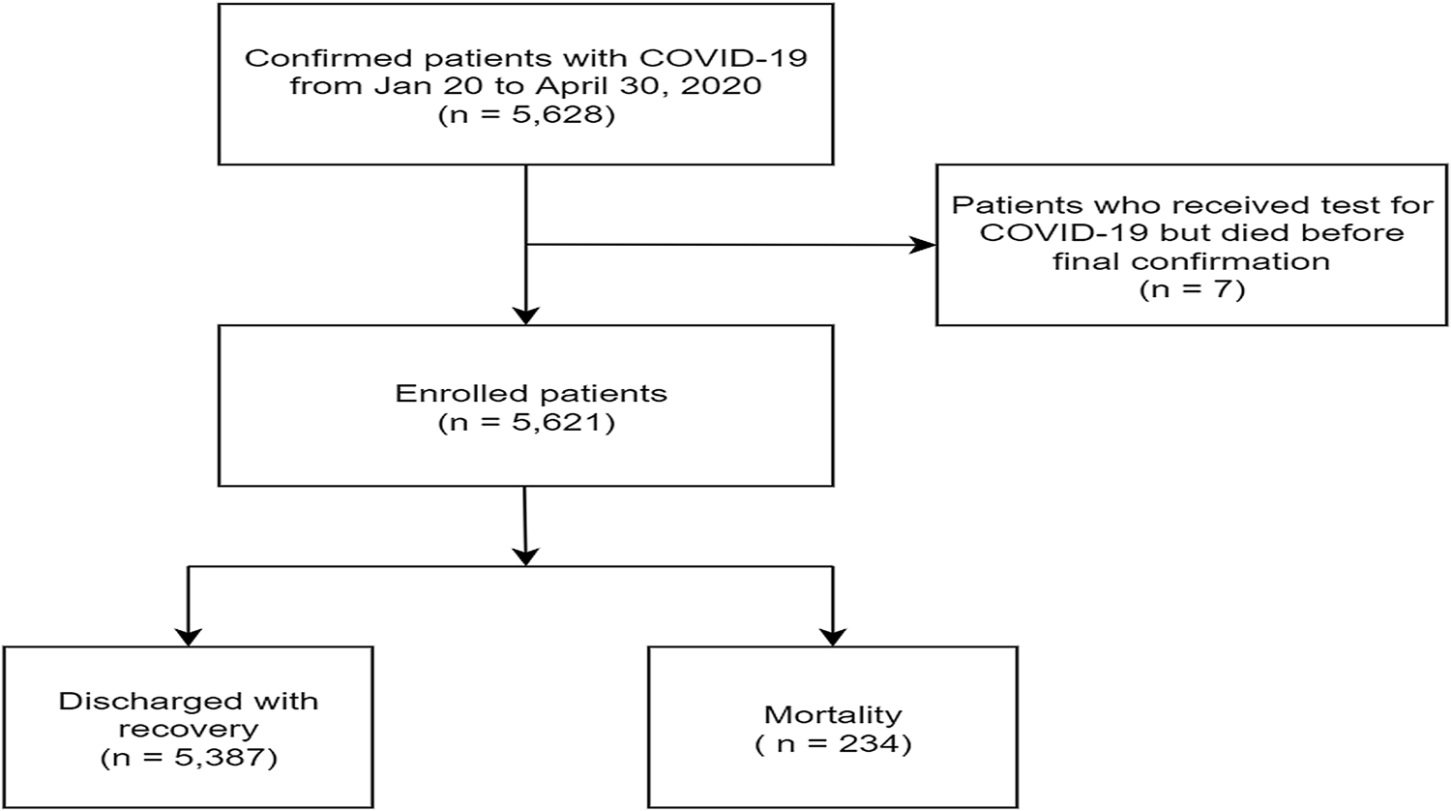
Flowchart of the study population.

A total of 5628 patients with confirmed COVID-19 were recorded in the KDCA registry during the study period. After excluding 7 patients who underwent laboratory tests for COVID-19 but died before the final confirmation of SARS-CoV-2 [using real-time reverse transcription polymerase chain reaction (RT-PCR) methods], we included 5621 patients in the final analysis (Fig. 1). Of the 5621 patients, 5387 were discharged while 234 died in the hospital (Table 1 and Fig. 1). The most common age group was 50–59 years, followed by 20–29 and 60–69 years. Of the 5621 patients, 2317 (41.2%) were men. Comorbidities were present in nearly half of the patients, including hypertension (21.3%), diabetes (12.3%), dementia (4.0%), chronic cardiac disease (3.2%), and malignancy (2.5%). The most common symptoms at presentation were cough and sputum, followed by fever and headache. Overall, the median isolation period was 24 days [interquartile range (IQR) 18–32]. The median survival (isolation) period among non-survivors was 11 days (IQR 6–21) after confirmation of COVID-19. Comparisons of clinical and epidemiological characteristics between survivors and non-survivors are shown in Table 1. Supplementary Table 1 presents the levels of complications that occurred during the isolation period among all patients. Patients who had no limitation of activity were 4482 (79.7%) while those requiring oxygen supply was 809 (14.4%).

**Table 1:** Baseline characteristics of COVID-19 patients in Korea.

Variables	Total (n = 5621)	Survivors (n = 5387)	Non-survivors (n = 234)	*p* value
**Age; years**				< 0.001
0–9	66	66 (100.0%)	0 (0.0%)	
10–19	206	206 (100.0%)	0 (0.0%)	
20–29	1119	1119 (100.0%)	0 (0.0%)	
30–39	564	562 (99.6%)	2 (0.4%)	
40–49	742	740 (99.7%)	2 (0.3%)	
50–59	1145	1131 (98.8%)	14 (1.2%)	
60–69	914	882 (96.5%)	32 (3.5%)	
70–79	542	472 (87.1%)	70 (12.9%)	
≥ 80	323	209 (64.7%)	114 (35.3%)	
Male, n (%)	2317 (41.2%)	2193 (40.7%)	124 (53.0%)	< 0.001
Isolation period, day	24 [18–32]	24 [18–32]	11 [6–21]	< 0.001
Obesity (BMI ≥ 25)	1260 (22.4%)	1216 (22.6%)	44 (18.8%)	< 0.001
Heart rate	85.9 ± 15.0	85.7 ± 14.8	89.5 ± 20.0	0.005
Body temperature	36.9 ± 0.6	36.9 ± 0.5	37.1 ± 0.8	0.002
**Symptoms, n (%)**				
Fever	1303 (23.2%)	1212 (22.5%)	91 (38.9%)	< 0.001
Cough	2340 (41.6%)	2260 (42.0%)	80 (34.2%)	0.021
Sputum	1618 (28.8%)	1547 (28.7%)	71 (30.3%)	0.648
Sore throat	880 (15.7%)	868 (16.1%)	12 (5.1%)	< 0.001
Rhinorrhoea	621 (11.0%)	615 (11.4%)	6 (2.6%)	< 0.001
Myalgia	925 (16.5%)	905 (16.8%)	20 (8.5%)	0.001
Fatigue/malaise	234 (4.2%)	217 (4.0%)	17 (7.3%)	0.024
Dyspnoea	663 (11.8%)	553 (10.3%)	110 (47.0%)	< 0.001
Headache	967 (17.2%)	954 (17.7%)	13 (5.6%)	< 0.001
Altered mentality	32 (0.6%)	12 (0.2%)	20 (8.5%)	< 0.001
Nausea/vomiting	244 (4.3%)	228 (4.2%)	16 (6.8%)	0.081
Diarrhoea	518 (9.2%)	500 (9.3%)	18 (7.7%)	0.477
**Comorbidity, n (%)**				
DM	689 (12.3%)	593 (11.0%)	96 (41.0%)	< 0.001
HTN	1199 (21.3%)	1057 (19.6%)	142 (60.7%)	< 0.001
Heart failure	58 (1.0%)	41 (0.8%)	17 (7.3%)	< 0.001
Chronic cardiac Ds	179 (3.2%)	153 (2.9%)	26 (11.1%)	< 0.001
Asthma	128 (2.3%)	115 (2.1%)	13 (5.6%)	0.001
COPD	40 (0.7%)	32 (0.6%)	8 (3.4%)	< 0.001
CKD	55 (1.0%)	39 (0.7%)	16 (6.8%)	< 0.001
Malignancy	143 (2.5%)	123 (2.3%)	20 (8.5%)	< 0.001
Chronic liver Ds	82 (1.5%)	76 (1.5%)	6 (2.6%)	0.310
Rheumatic or autoimmune Ds	38 (0.7%)	35 (0.7%)	3 (1.3%)	0.517
Dementia	224 (4.0%)	149 (2.9%)	75 (32.1%)	< 0.001
ICU admission	187 (3.3%)	105 (2.0%)	82 (35.0%)	< 0.001
**Laboratory data**				
WBC	5690 [4442–7170]	5660 [4430–7090]	6440 [4780–9680]	< 0.001
Lymphocyte	28.9 [21.3–36.5]	29.6 [22.5–36.9]	12.2 [7.2–19.9]	< 0.001
Haemoglobin	13.3 [12.2–14.4]	13.3 [12.3–14.5]	11.7 [10.6–12.8]	< 0.001
Haematocrit	39.2 [36.4–42.4]	39.4 [36.6–42.5]	[30.5–39.0]	< 0.001
Platelet	228k [180k–284k]	232k [183k–286k]	168k [130k–223k]	< 0.001

*COVID-19* coronavirus disease 2019, *BMI* body mass index, *DM* diabetes mellitus, *HTN* hypertension, *Ds* disease, *COPD* chronic obstructive pulmonary disease, *CKD* chronic kidney disease, *ICU* intensive care unit, *WBC* white blood cell.

### Kaplan–Meier survival curves according to age and sex

**Figure 2:**
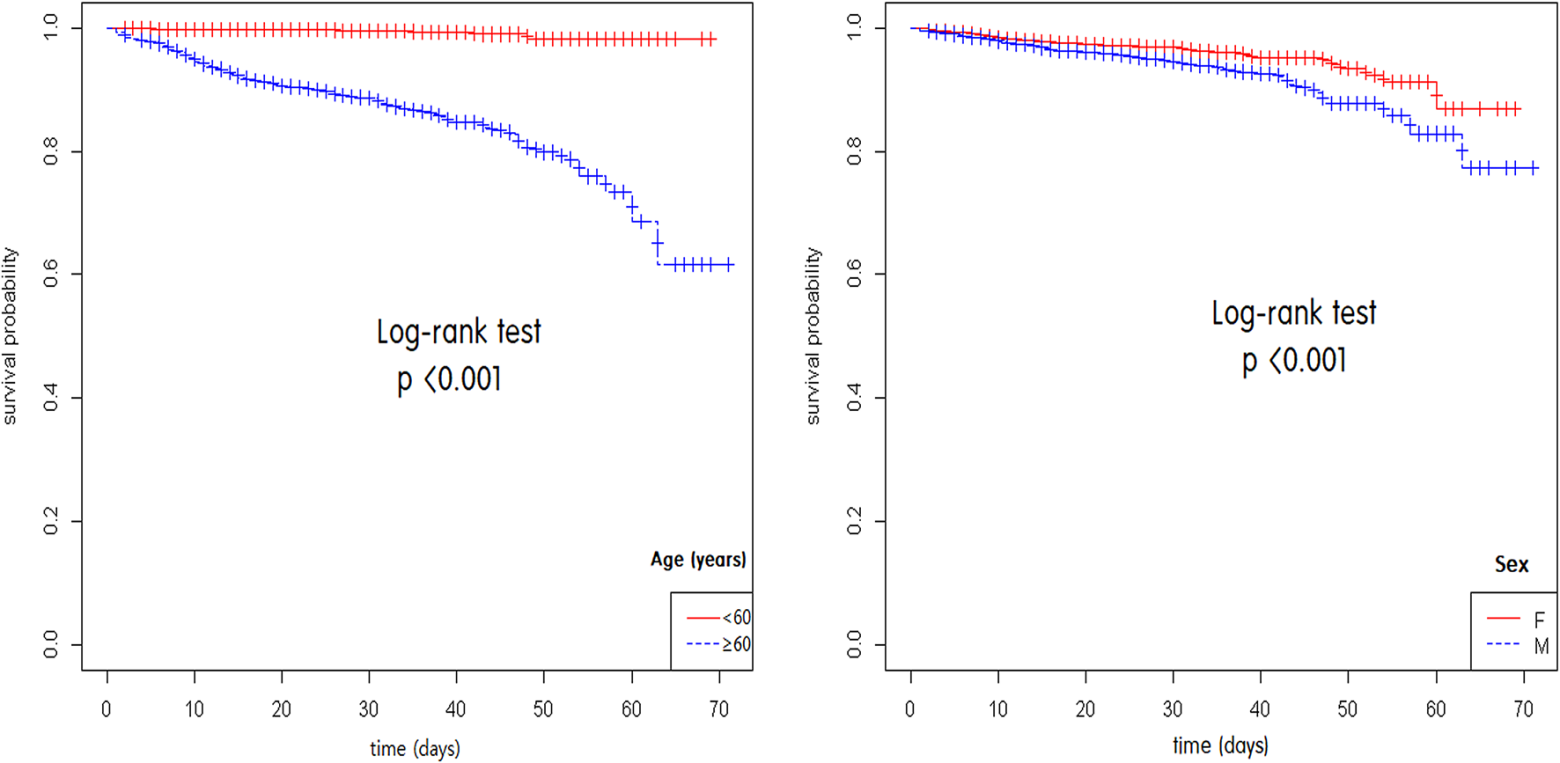
Kaplan–Meier survival curves stratified by age (**a**) and sex (**b**).

Mortality was significantly higher in elderly patients (age ≥ 65 years) than in younger patients (age < 65 years) (log-rank test, *p* < 0.001) (Fig. 2a) and in male patients than in female patients (log-rank test, *p* < 0.001) (Fig. 2b).

### Factors associated with mortality and fatal adverse outcomes

#### Logistic regression analysis

In univariable logistic regression analysis, the odds of mortality were higher in patients aged ≥ 60 years, and those with altered mentality, dementia, heart failure, and chronic kidney disease (Table 2). Sex, isolation period, obesity, heart rate, body temperature, fever, dyspnoea, diabetes, hypertension, chronic cardiac disease, asthma, chronic obstructive pulmonary disease, malignancy, and leucocytosis, were also associated with mortality (Table 2). Multivariable logistic regression analysis results showed increasing odds of mortality with age ≥ 60 years, short isolation period, dyspnoea, altered mentality, diabetes, malignancy, dementia, intensive care unit (ICU) admission, and lower levels of lymphocyte, haemoglobin, or platelet (Table 2). The multiple regression equation including all the potential variables effectively predicted mortality in COVID-19 patients [area under the curve (AUC) = 0.979, 95% confidence interval (CI) 0.964–0.993].

**Table 2:** Logistic regression analysis for mortality among COVID-19 patients.

	Univariable OR (95% CI)	*p* value	Multivariable OR (95% CI)	*p* value
**Age**				
0–59 years	1 (Reference group)		1 (Reference group)	
≥ 60 years	29.359 (18.627–49.365)	< 0.001	11.685 (4.655–34.150)	< 0.001
**Sex**				
Female	1 (Reference group)		1 (Reference group)	
Male	1.642 (1.263–2.137)	< 0.001	1.818 (0.914–3.646)	0.089
Isolation period	0.877 (0.861–0.893)	< 0.001	0.892 (0.864–0.918)	< 0.001
Obesity (BMI ≥ 25)	0.913 (0.885–0.937)	< 0.001	0.883 (0.751–1.054)	0.093
Heart rate	1.016 (1.007–1.024)	< 0.001	1.017 (0.999–1.036)	0.074
Body temperature	1.603 (1.289–1.980)	< 0.001		
**Symptom**				
Fever	2.190 (1.666–2.684)	< 0.001		
Cough	0.718 (0.543–0.943)	0.018		
Sore throat	0.281 (0.524–0.728)	< 0.001		
Rhinorrhoea	0.204 (0.080–0.421)	< 0.001	0.078 (0.010–0.399)	0.006
Myalgia	0.462 (0.282–0.716)	0.001		
Fatigue/malaise	1.865 (1.079–3.023)	0.017		
Dyspnoea	7.748 (5.902–10.162)	< 0.001	4.178 (2.182–8.101)	< 0.001
Headache	0.273 (0.148–0.460)	< 0.001		
Altered mentality	41.83 (20.46–89.182)	< 0.001	25.181 (2.307–242.638)	0.006
**Past medical history**				
DM	5.620 (4.264–7.382)	< 0.001	2.728 (1.439–5.220)	0.002
HTN	6.318 (4.829–8.306)	< 0.001		
Heart failure	10.209 (5.569–17.950)	< 0.001	2.937 (0.683–11.550)	0.133
Chronic cardiac Ds	4.26 (2.695–6.496)	< 0.001		
Asthma	2.695 (1.428–4.682)	< 0.001		
COPD	5.920 (2.515–12.379)	< 0.001	3.504 (0.633–1.616)	0.126
CKD	10.059 (5.389–17.949)	< 0.001		
Malignancy	3.997 (2.378–6.393)	< 0.001	4.127 (1.258–12.561)	0.015
Dementia	15.541 (11.263–21.346)	< 0.001	11.612 (4.964–27.855)	< 0.001
ICU admission	26.994 (19.377–37.580)	< 0.001	21.327 (10.044–47.529)	< 0.001
**Laboratory data**				
WBC	1.000 (1.000–1.000)	< 0.001		
Lymphocyte	0.863 (0.847–0.877)	< 0.001	0.901 (0.869–0.931)	< 0.001
Haemoglobin	0.638 (0.593–0.685)	< 0.001	0.701 (0.590–0.829)	< 0.001
Haematocrit	0.850 (0.828–0.872)	< 0.001		
Platelet	0.999 (0.999–0.999)	< 0.001	0.999 (0.999–0.999)	< 0.001

*COVID-19* coronavirus disease 2019, *OR* odds ratio, *CI* confidence interval, *BMI* body mass index, *DM* diabetes, *HTN* hypertension, *Ds* disease, *COPD* chronic obstructive pulmonary disease, *CKD* chronic kidney disease, *ICU* intensive care unit, *WBC* white blood cell.

Univariate analysis showed that the odds of fatal adverse outcomes were higher in patients aged ≥ 60 years, and those with altered mentality, dementia, heart failure, and chronic kidney disease (Table 3). Sex, isolation period, obesity, heart rate, body temperature, fever, dyspnoea, diabetes, hypertension, chronic cardiac disease, asthma, chronic obstructive pulmonary disease, malignancy, and leucocytosis, were also associated with fatal adverse outcomes (Table 3). Multivariable logistic regression analysis showed increasing odds of fatal adverse outcomes associated with age ≥ 60 years, short isolation period, dyspnoea, altered mentality, diabetes, hypertension, malignancy, dementia, ICU admission, and lower levels of lymphocytes, haemoglobin, or platelets (Table 3).

**Table 3:** Logistic regression analysis of factors associated with fatal adverse outcomes (invasive mechanical ventilation, multi-organ failure, ECMO, and death) among COVID-19 patients.

	Univariable OR (95% CI)	*p* value	Multivariable OR (95% CI)	*p* value
**Age**				
0–59 years	1 (Reference group)		1 (Reference group)	
≥ 60 years	23.722 (15.974–36.855)	< 0.001	3.891 (1.858–8.686)	< 0.001
**Sex**				
Female	1 (Reference group)		1 (Reference group)	
Male	1.675 (1.307–2.148)	< 0.001	1.803 (0.970–3.380)	0.063
Isolation period	0.921 (0.907–0.935)	< 0.001	0.951 (0.929–0.971)	< 0.001
Obesity (BMI ≥ 25)	0.924 (0.896–0.946)	< 0.001	0.892 (0.760–1.065)	0.098
Heart rate	1.014 (1.006–1.022)	< 0.001	1.004 (0.987–1.022)	0.661
Body temperature	1.707 (1.394–2.078)	< 0.001		
**Symptom**				
Fever	2.207 (1.705–2.844)	< 0.001		
Cough	0.725 (0.557–0.937)	0.015		
Sore throat	0.270 (0.146–0.454)	< 0.001	0.324 (0.082–1.052)	0.082
Rhinorrhoea	0.242 (0.109–0.459)	< 0.001	0.098 (0.018–0.424)	0.004
Myalgia	0.519 (0.333–0.772)	0.002		
Fatigue/malaise	1.739 (1.023–2.783)	0.029		
Dyspnoea	8.894 (6.875–11.509)	< 0.001	0.463 (2.567–8.486)	< 0.001
Headache	0.319 (0.187–0.507)	< 0.001		
Altered mentality	56.381 (26.676–129.839)	< 0.001	145.008 (12.859–1538.967)	< 0.001
**Past medical history**				
DM	5.299 (4.072–6.872)	< 0.001	1.838 (1.000–3.365)	0.049
HTN	6.594 (5.110–8.547)	< 0.001	1.848 (1.027–3.351)	0.041
Heart failure	10.515 (5.870–18.227)	< 0.001	2.457 (0.632–8.502)	0.174
Chronic cardiac Ds	4.246 (2.746–6.360)	< 0.001		
Asthma	2.347 (1.245–4.069)	0.004		
COPD	6.030 (2.678–12.291)	< 0.001	4.771 (0.979–19.586)	0.039
CKD	10.461 (5.749–18.385)	< 0.001		
Malignancy	3.467 (2.067–5.533)	< 0.001	3.162 (0.999–9.225)	0.042
Dementia	13.256 (9.669–18.082)	< 0.001	7.924 (3.548–17.842)	< 0.001
ICU admission	38.837 (28.030–54.009)	< 0.001	25.041 (12.776–50.911)	< 0.001
**Laboratory data**				
WBC	1.0001 (1.0001–1.0002)	< 0.001		
Lymphocyte	0.859 (0.844–0.873)	< 0.001	0.904 (0.873–0.935)	< 0.001
Haemoglobin	0.651 (0.607–0.696)	< 0.001	0.670 (0.487–0.974)	0.022
Haematocrit	0.856 (0.835–0.877)	< 0.001		
Platelet	0.999 (0.999–0.999)	< 0.001	0.9999 (0.999–0.999)	< 0.001

*ECMO* extracorporeal membrane oxygenation, *COVID-19* coronavirus disease 2019, *OR* odds ratio, *CI* confidence interval, *BMI* body mass index, *DM* diabetes mellitus, *HTN* hypertension, *Ds* disease, *COPD* chronic obstructive pulmonary disease, *CKD* chronic kidney disease, *ICU* intensive care unit, *WBC* white blood cell.

#### Cox proportional hazards model

**Figure 3:**
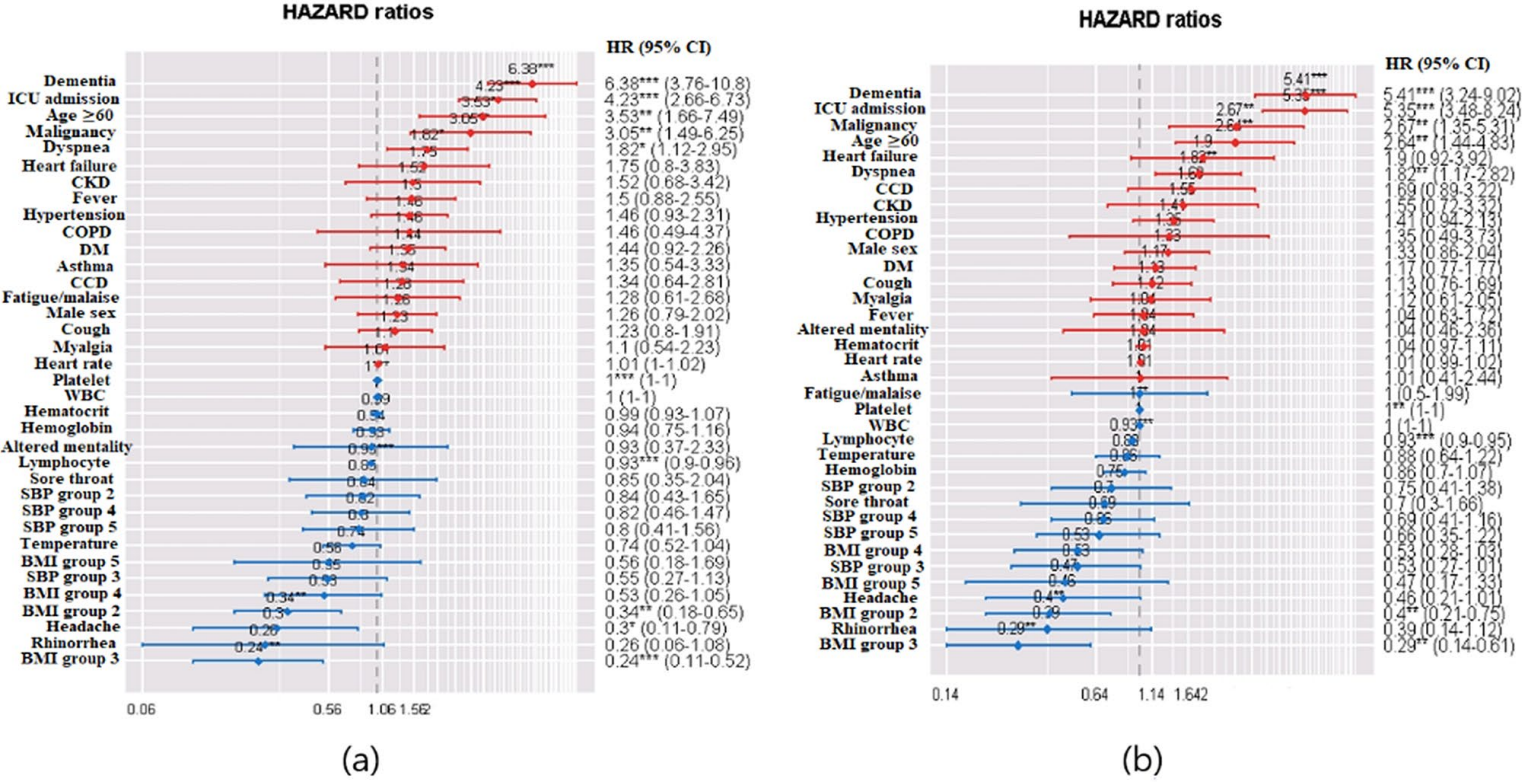
Hazards ratio plot in prediction of mortality (**a**) and fatal adverse outcomes (**b**).

The multivariable Cox proportional hazards model showed increasing hazards of mortality with dementia, ICU admission, age ≥ 60 years, malignancy, and dyspnoea (Table 4). The hazard ratio plot for mortality using the multivariable Cox proportional hazards model is presented in Fig. 3a. Body mass index (BMI) 18.5–22.9 or 23.0–24.9, headache, and higher levels of lymphocytes or platelets were associated with decreasing mortality hazards. The multivariable Cox proportional hazards model showed that increasing hazards of fatal adverse outcomes were associated with dementia, ICU admission, age ≥ 60 years, malignancy, and dyspnoea (Table 5). The hazard ratio plot for fatal adverse outcomes using the multivariable Cox proportional hazards model is presented in Fig. 3b. BMI, 18.5–22.9 or 23.0–24.9, and higher levels of lymphocytes or platelets were associated with decreasing hazards of fatal adverse outcomes.

**Table 4:** Cox proportional hazards model for mortality among COVID-19 patients.

	Univariable HR (95% CI)	*p* value	Multivariable HR (95% CI)	*p* value
**Age**				
0–59 years	1 (Reference group)		1 (Reference group)	
≥ 60 years	24.532 (15.163–39.716)	< 0.001	3.530 (1.664–7.485)	0.001
**Sex**				
Female	1 (Reference group)		1 (Reference group)	
Male	1.633 (1.263–2.111)	< 0.001	1.261 (0.786–2.024)	0.337
**BMI**				
< 18.5	1 (Reference group)		1 (Reference group)	
18.5–22.9	0.376 (0.213–0.665)	< 0.001	0.314 (0.178–0.653)	0.001
23.0–24.9	0.299 (0.155–0.577)	< 0.001	0.240 (0.110–0.522)	< 0.001
25.0–29.9	0.547 (0.305–0.979)	0.042	0.525 (0.263–1.051)	0.069
≥ 30.0	0.352 (0.129–0.962)	0.042	0.556 (0.183–1.690)	0.301
**SBP (mmHg)**				
< 120	1 (Reference group)		1 (Reference group)	
120–129	0.523 (0.333–0.822)	0.005	0.840 (0.427–1.654)	0.614
130–139	0.636 (0.413–0.980)	0.040	0.547 (0.265–1.127)	0.102
140–159	1.023 (0.721–1.453)	0.898	0.823 (0.460–1.473)	0.512
≥ 160	1.526 (1.010–2.306)	0.045	0.801 (0.410–1.564)	0.515
Heart rate	1.016 (1.007–1.024)	< 0.001	1.007 (0.995–1.020)	0.239
Body temperature	1.494 (1.216–1.837)	< 0.001	0.736 (0.520–1.040)	0.082
**Symptom**				
Fever	2.019 (1.551–2.627)	< 0.001	1.501 (0.882–2.554)	0.135
Cough	0.673 (0.513–0.881)	0.004	1.232 (0.796–1.908)	0.349
Sore throat	0.287 (0.161–0.513)	< 0.001	0.846 (0.351–2.041)	0.710
Rhinorrhoea	0.206 (0.092–0.463)	< 0.001	0.256 (0.060–1.082)	0.064
Myalgia	0.445 (0.281–0.703)	< 0.001	1.100 (0.544–2.225)	0.791
Fatigue/Malaise	1.742 (1.062–2.851)	0.028	1.283 (0.614–2.679)	0.507
Dyspnoea	6.392 (4.94–8.265)	< 0.001	1.823 (1.125–2.954)	0.015
Headache	0.274 (0.157–0.480)	< 0.001	0.300 (0.113–0.794)	0.015
Altered mentality	19.772 (12.461–31.353)	< 0.001	0.931 (0.372–2.332)	0.879
**Past medical history**				
DM	4.656 (3.585–6.049)	< 0.001	1.439 (0.917–2.259)	0.114
HTN	5.423 (4.169–7.054)	< 0.001	1.462 (0.927–2.306)	0.102
Heart failure	7.693 (4.691–12.621)	< 0.001	1.755 (0.804–3.832)	0.158
Chronic cardiac Ds	3.563 (2.368–5.361)	< 0.001	1.344 (0.642–2.814)	0.433
Asthma	2.384 (1.362–4.173)	0.002	1.346 (0.544–3.332)	0.521
COPD	4.484 (2.211–9.093)	< 0.001	1.457 (0.486–4.367)	0.501
CKD	7.191 (4.316–11.982)	< 0.001	1.519 (0.676–3.417)	0.312
Malignancy	3.389 (2.141–5.364)	< 0.001	3.054 (1.494–6.245)	0.002
Dementia	11.34 (8.608–14.931)	< 0.001	6.376 (3.763–10.802)	< 0.001
ICU admission	14.810 (11.281–19.432)	< 0.001	4.233 (2.661–6.734)	< 0.001
**Laboratory data**				
WBC	1.000 (1.000–1.000)	< 0.001	1.0000 (0.9999–1.0001)	0.183
Lymphocyte	0.875 (0.862–0.889)	< 0.001	0.930 (0.903–0.956)	< 0.001
Haemoglobin	0.676 (0.637–0.719)	< 0.001	0.936 (0.754–1.162)	0.547
Haematocrit	0.874 (0.857–0.891)	< 0.001	0.994 (0.926–1.066)	0.866
Platelet	0.999 (0.999–0.999)	< 0.001	1.000 (0.999–1.000)	< 0.001

*COVID-19* coronavirus disease 2019, *HR* hazard ratio, *CI* confidence interval, *BMI* body mass index, *SBP* systolic blood pressure, *DM* diabetes, *HTN* hypertension, *Ds* disease, *COPD* chronic obstructive pulmonary disease, *CKD* chronic kidney disease, *ICU* intensive care unit, *WBC* white blood cell.

**Table 5:** Cox proportional hazards model for fatal adverse outcomes among COVID-19 patients.

	Univariable HR (95% CI)	*p* value	Multivariable HR (95% CI)	*p* value
**Age**				
0–59 years	1 (Reference group)		1 (Reference group)	
≥ 60 years	18.942 (12.541–28.623)	< 0.001	2.640 (1.443–4.830)	0.002
**Sex**				
Female	1 (Reference group)		1 (Reference group)	
Male	1.649 (1.294–2.101)	< 0.001	1.326 (0.863–2.038)	0.198
**BMI**				
< 18.5	1 (Reference group)		1 (Reference group)	
18.5–22.9	0.454 (0.260–0.793)	0.006	0.399 (0.213–0.746)	0.004
23.0–24.9	0.374 (0.200–0.703)	0.002	0.293 (0.141–0.605)	< 0.001
25.0–29.9	0.679 (0.385–1.196)	0.180	0.534 (0.277–1.033)	0.062
≥ 30.0	0.434 (0.176–1.071)	0.070	0.472 (0.167–1.335)	0.157
**SBP (mmHg)**				
< 120	1 (Reference group)		1 (Reference group)	
120–129	0.504 (0.330–0.770)	0.002	0.751 (0.408–1.382)	0.357
130–139	0.613 (0.408–0.921)	0.018	0.526 (0.273–1.014)	0.055
140–159	1.000 (0.720–1.390)	0.999	0.686 (0.406–1.159)	0.159
≥ 160	1.457 (0.987–2.153)	0.058	0.657 (0.354–1.219)	0.183
Heart rate	1.014 (1.006–1.022)	< 0.001	1.005 (0.995–1.016)	0.336
Body temperature	1.534 (1.269–1.855)	< 0.001	0.884 (0.639–1.223)	0.457
**Symptom**				
Fever	1.957 (1.526–2.509)	< 0.001	1.041 (0.631–1.718)	0.874
Cough	0.659 (0.510–0.850)	0.001	1.131 (0.760–1.685)	0.543
Sore throat	0.278 (0.159–0.486)	< 0.001	0.699 (0.295–1.656)	0.416
Rhinorrhoea	0.245 (0.121–0.495)	< 0.001	0.391 (0.137–1.118)	0.080
Myalgia	0.492 (0.326–0.743)	< 0.001	1.118 (0.609–2.051)	0.719
Fatigue/malaise	1.633 (1.012–2.637)	0.045	1.000 (0.502–1.993)	0.999
Dyspnoea	6.859 (5.383–8.739)	< 0.001	1.815 (1.167–2.823)	0.008
Headache	0.323 (0.198–0.529)	< 0.001	0.460 (0.210–1.010)	0.052
Altered mentality	19.548 (12.714–30.107)	< 0.001	1.043 (0.462–2.358)	0.919
**Past medical history**				
DM	4.121 (3.211–5.287)	< 0.001	1.167 (0.770–1.769)	0.467
HTN	5.360 (4.178–6.875)	< 0.001	1.414 (0.940–2.126)	0.096
Heart failure	7.736 (4.843–12.361)	< 0.001	1.900 (0.920–3.924)	0.083
Chronic cardiac Ds	3.377 (2.293–4.973)	< 0.001	1.690 (0.887–3.222)	0.111
Asthma	2.060 (1.178–3.601)	0.011	1.006 (0.414–2.444)	0.990
COPD	4.014 (2.049–7.863)	< 0.001	1.347 (0.487–3.726)	0.566
CKD	6.087 (3.694–10.031)	< 0.001	1.550 (0.723–3.324)	0.261
Malignancy	2.901 (1.835–4.582)	< 0.001	2.673 (1.346–5.310)	0.005
Dementia	9.746 (7.455–12.740)	< 0.001	5.406 (3.241–9.016)	< 0.001
ICU admission	16.391 (12.742–21.104)	< 0.001	5.355 (3.482–8.236)	< 0.001
**Laboratory data**				
WBC	1.000 (1.000–1.000)	< 0.001	1.000 (1.000–1.000)	0.406
Lymphocyte	0.875 (0.862–0.887)	< 0.001	0.927 (0.902–0.952)	< 0.001
Haemoglobin	0.690 (0.651–0.731)	< 0.001	0.863 (0.697–1.067)	0.173
Haematocrit	0.880 (0.863–0.897)	< 0.001	1.037 (0.966–1.113)	0.317
Platelet	0.999 (0.999–0.999)	< 0.001	1.000 (1.000–1.000)	0.006

*COVID-19* coronavirus disease 2019, *HR* hazard ratio, *CI* confidence interval, *BMI* body mass index, *SBP* systolic blood pressure, *DM* diabetes, *HTN* hypertension, *Ds* disease, *COPD* chronic obstructive pulmonary disease, *CKD* chronic kidney disease, *ICU* intensive care unit, *WBC* white blood cell.

## Discussion

To our knowledge, this is one of the largest nationwide cohort studies in COVID-19 patients with definite clinical outcomes. This nationwide retrospective cohort study identified the risk factors of mortality and fatal adverse outcomes among patients with laboratory-confirmed COVID-19 in Korea. In particular, dementia, ICU admission, age ≥ 60 years, malignancy, and dyspnoea, were associated with higher odds of mortality. In addition, BMI 18.5–22.9 or 23.0–24.9, and higher levels of lymphocytes or platelets, were associated with lower odds of poor clinical outcomes.

The present study showed that older age (≥ 60 years) was associated with mortality and fatal adverse outcomes in patients with COVID-19. Although individuals > 65 years comprise 17% of the total population in the United States, they account for 31% of COVID-19 patients, 45% of hospitalized patients, 53% of those on intensive care unit admission, and 80% of those that died due to COVID-19^14^. This suggests that older individuals are susceptible to developing COVID-19 and have poor clinical outcomes compared with the general population. Our results showed that individuals ≥ 60 accounted for 32% of COVID-19 and 92% of deaths due to COVID-19. In accordance with our study, recent studies reported that older age is significantly associated with mortality among COVID-19 patients^9,10^.

Previous studies reported that male sex is significantly associated with mortality^9,15^. Our data showed that although mortality was significantly higher in male patients than in female patients in the Kaplan–Meier survival curve analysis, male sex was not a significant risk factor for mortality in the Cox proportional hazards model. We recommend a larger worldwide cohort study to explore this discrepancy between our results and those of previous studies.

The risk of serious disease and mortality in COVID-19 increases with the presence of comorbidities^16–20^. Our data showed malignancy as an independent risk factor for mortality in the multivariable Cox proportional hazards model with a hazard ratio (HR) of 3.054. According to a recent study from China, cancer patients with COVID-19 had 3.5 times higher risk of requiring ICU admission or mechanical ventilation, compared to the general population^21^. Cancer patients are generally immunocompromised and are at higher risk of COVID-19-related fatal events in comparison to the cancer-free population^21,22^.

Our study also showed that dementia was associated with mortality and fatal adverse outcomes. Some reasons can be proposed to explain our results. First, most of the patients suffering dementia are old and have other comorbidities that could result in poor clinical outcomes^23^. Second, people with dementia would require the support of dementia caregivers in order to keep preventive and healthcare measures. However, this COVID-19 pandemic has made the availability of caregivers limited^24^. Third, persistent inflammatory state of dementia patients may increase peripheral blood white blood cells and neutrophil counts, which was associated with higher mortality from COVID-19^25^. Fourth, the ApoE e4 genotype, which were associated with patients with dementia^26^, can increase the risk of having acute respiratory distress syndrome in COVID-19 patients. Similar to our data, a previous study reported that mortality due to COVID-19 was significantly higher in elderly patients with dementia^27^. Another study reported an in-hospital mortality of 61% among hospitalized patients with dementia and COVID-19^28^. In our study, mortality during the isolation period was 33.5% among patients with COVID-19. Two large cohort studies from the United Kingdom showed that pre-existing dementia was a significant predictor of COVID-19 mortality^11,20^. Furthermore, a recent study, including global big data on dementia and COVID-19 in 185 countries revealed a significant correlation between dementia burden and COVID-19-related death^29^.

In the present study, although diabetes mellitus (DM) was not a predictor of mortality in the Cox proportional hazards model, it was associated with mortality and fatal adverse outcomes in multivariable logistic regression analysis. Some case series reported that patients with pre-existing DM are vulnerable to infection and are at a higher risk of death from COVID-19^5,15,30,31,32^. According to a recent meta-analysis, diabetes in patients with COVID-19 is associated with a two-fold increase in mortality as well as severity of COVID-19, as compared to those in non-diabetics^33^. Similar to the prior meta-analysis, our results showed that DM was associated with increased odds of mortality of 2.728 and fatal adverse outcomes of 1.838 in multivariable logistic regression analysis.

Our study demonstrated that dyspnoea and ICU admission were associated with mortality and fatal adverse outcomes in both the multivariable logistic regression analysis and Cox proportional hazards model. Data from urban communities in the United States revealed that patients admitted to the ICU had a higher incidence of respiratory failure requiring invasive mechanical ventilation and mortality compared with patients in the general ward^9^. Another study showed that dyspnoea at presentation was associated with hospitalization and the need for ICU management^9^. According to a recent meta-analysis, dyspnoea was the only symptom predictive of severe COVID-19 and ICU admission^19^. Similar to the previous meta-analysis, our study showed that dyspnoea was the only symptom predictive of fatal adverse outcomes.

A recent study using a systemic review and meta-analysis showed that hematologic and inflammatory markers such as procalcitonin, C-reactive protein, D-dimer, and lactate dehydrogenase and decreased albumin could help predict severe outcomes in COVID-19^34^. Our study showed that lower levels of lymphocytes or platelets were associated with increasing hazards of mortality and fatal adverse outcomes. COVID-19 patients with low levels of lymphocyte and platelets should be monitored closely to minimize the risk of progression to severe disease.

The present study has some limitations. First, due to the retrospective nature of the study design and data extraction procedure, some laboratory data were missing or unavailable. Therefore, their influence might have been underestimated in predicting mortality and fatal adverse outcomes. Second, our data did not include information regarding radiologic findings, which might reveal prognostic factors^35^. Third, although treatment strategy can have a significant impact on clinical outcome^36–38^, we assumed that enrolled patients all had adequate and timely management. Fourth, although our study included over 5600 patients with COVID-19 from Korea, there was a lack of time-series analysis for potential variables. Fifth, by excluding patients still under isolation as of April 30, 2020, the case fatality ratio in our data could not reflect the true mortality of COVID-19. Sixth, our study did not include data regarding the status of therapy for each comorbidity. For instance, metformin use was associated with reduced mortality^39^, but statin use was not^40^.

## Conclusions

This nationwide cohort study showed that dementia, ICU admission, age ≥ 60 years, malignancy, and dyspnoea were strong predictors of death and fatal adverse outcomes in COVID-19 patients. The multiple regression equation including clinical and epidemiological variables predicted mortality in such patients. These findings can help clinicians to identify COVID-19 patients with poor prognosis.

## Methods

### Study design and population

This retrospective cohort study included a nationwide cohort data of COVID-19 patients collected by KDCA, Korea. All patients who were diagnosed with COVID-19 were screened, and those who died or were discharged between January 20, 2020 (i.e., when the first patients were diagnosed in Korea), and April 30, 2020, were included in the present study. Since KDCA is a governmental institution responsible for surveillance and control of COVID-19 in Korea, our study enrolled all the patients who were diagnosed with COVID-19 and had a definite clinical outcome (discharged or dead) at the early stage of the outbreak.

This study was conducted according to the principles of the Declaration of Helsinki and was approved by the Institutional Review Board (IRB) of the Korea University Ansan Hospital (IRB number 2020AS0245). The requirement for informed consent was waived by the IRB because of the retrospective nature of the study.

### Data source

Clinical, epidemiological, demographic, laboratory, and outcome data were collected by KDCA since the diagnosis of the first patients with COVID-19 on January 20, 2020. All data were checked by the physicians and the KDCA preventive medicine experts. We obtained permission to access the encoded KDCA server for three weeks to extract and analyse the data during the limited period. These data cannot be shared publicly according to the KDCA directive, which prohibits researchers from such. KCDA provided anonymised clinical data for the public interest.

### Laboratory results

For the laboratory confirmation of SARS-CoV-2, real-time RT-PCR was performed using throat-swab specimens. The criteria for discharge were two negative throat-swab samples for SARS-CoV-2 RNA, absence of fever for at least 3 days, definite improvement of pneumonia on chest computed tomography, and stable vital signs. Routine blood examination included a complete blood count.

### Definitions

We defined fever as an axillary temperature of at least 37.5 °C. Fatal adverse outcomes were defined as at least one of invasive mechanical ventilation, multi-organ failure (MOF), ECMO, or death during the isolation period. In the present study, MOF refers to altered function in two or more organ systems after the diagnosis of COVID-19.

### Statistical analysis

Statistical analyses were performed using R 4.0.2 (the R Foundation for Statistical Computing, Vienna, Austria). For all analyses, a 2-tailed test with a *p* value < 0.05 was considered statistically significant. Continuous variables were presented as median (IQR) or mean [standard deviation (SD)], and categorical variables were presented as frequencies (percentages). We used the t test, Mann–Whitney U test, χ^2^ test, or Fisher’s exact test to compare differences between survivors and non-survivors, as appropriate. The risk factors for death and fatal adverse outcomes in patients were determined using univariable and multivariable logistic regression models and presented as odds ratios (ORs) and 95% CIs. We excluded variables from the univariable logistic regression analysis if their between-group differences (the t test, Mann–Whitney U test, χ^2^ test, or Fisher’s exact test) were not significant. We considered variables with *p* ≤ 0.05 in the univariate analysis as candidate variables for the multivariable analysis. Kaplan–Meier curves and log-rank tests for age and sex were also performed for survival analyses. To explore whether potential variables were independently associated with shortened survival, we used univariable and multivariable Cox proportional-hazards models to calculate the HR and 95% CI for each variable.

## Supplementary information

Supplementary Information.

## Data Availability

The data that support the findings of this study are available from KDCA but restrictions apply to the availability of these data, which were used under license for the current study, and so are not publicly available. Data are however available from the authors upon reasonable request and with permission of KDCA. Data cannot be shared publicly according to the KDCA directive, which prohibits researchers from such. KCDA provided anonymised clinical data for the public interest.
