# Supplementary material for: Clinical course and risk factors of fatal adverse outcomes in COVID-19 patients in Korea: a nationwide retrospective cohort study
Source: Sci Rep. 2021 May 12;11:10066. doi: 10.1038/s41598-021-89548-y (PMC8115137; doi:10.1038/s41598-021-89548-y)
Supplement: Supplementary file 1 — Supplementary Information. [file 41598_2021_89548_MOESM1_ESM.docx]

**Clinical course and risk factors of fatal adverse outcomes in COVID-19 patients in Korea: a nationwide retrospective cohort study**

**Juhyun Song^1^, Dae Won Park^3^, Jae-hyung Cha^2^, Hyeri Seok^3^, Joo Yeong Kim^1^, Jonghak Park^1^ & Hanjin Cho^1^***

^1^Department of Emergency Medicine, Korea University Ansan Hospital, Ansan, Republic of Korea.

^2^Medical Science Research Centre, Korea University Ansan Hospital, Ansan, Republic of Korea.

^3^Division of Infectious Diseases, Department of Internal Medicine, Korea University Ansan Hospital, Ansan, Republic of Korea.

*email: chohj327@korea.ac.kr

**Supplementary Table 1.** Levels of complications occurring during the isolation period among COVID-19 patients.

| **Levels of complications during isolation period** | **Number of patients (n = 5,621)** |
| --- | --- |
| No limitation of activity | 4,482 |
| Limited activity but no oxygen supply required | 330 |
| Oxygen supply with nasal prong required | 469 |
| Oxygen supply with facial mask required | 43 |
| Non-invasive mechanical ventilation | 33 |
| Invasive mechanical ventilation | 19 |
| Multi-organ failure or ECMO | 11 |
| Death | 234 |

COVID-19, coronavirus disease 2019; ECMO, extracorporeal membrane oxygenation
